# Severe anemia may not be a contraindication to debridement in a Jehovah’s witness patient with necrotizng fasciitis of the lower extremity – A case report

**DOI:** 10.1016/j.ijscr.2019.08.031

**Published:** 2019-09-04

**Authors:** Chinwe Ogedegbe, Jayson Fernando, Sanjeev Kaul

**Affiliations:** a Department of Trauma and Critical Care Surgery, Hackensack University Medical Center, 30 Prospect Ave, Hackensack, NJ 07601, USA; b Department of Emergency Medicine, Hackensack University Medical Center, Hackensack, NJ, USA; c St. George’s University School of Medicine, Grenada, West Indies

**Keywords:** Necrotizing fasciitis, Jehovah’s Witness, Severe anemia, Case report

## Abstract

**Introduction:**

Necrotizing fasciitis is a severe soft tissue infection characterized by rapidly progressing necrosis involving the fascia and subcutaneous tissue. Necrotizing fasciitis of the lower extremity in a Jehovah’s Witness patient in the setting of severe anemia and systemic sepsis is uncommon.

**Case presentation:**

A 62-year-old man of Jehovah’s Witness faith with a history of alcohol use disorder and uncontrolled diabetes mellitus initially presented with a non-healing diabetic foot ulcer, subsequently developed sepsis and necrotizing fasciitis. He underwent an above the knee amputation and was transferred to our institution’s Surgical Intensive Care Unit for further management. The patient presented in critical condition with a hemoglobin of 4.7 g/dL and progression of necrotizing fasciitis of the lower extremity stump. He underwent revision amputation and numerous excisional debridements along with IV antibiotics, epoetin alfa, and iron sucrose. He successfully recovered with minimal blood loss and was discharged with a hemoglobin of 8 g/dL.

**Discussion:**

This case highlights some of the challenges involved in managing necrotizing fasciitis. The conversation with the Jehovah’s Witness patient in a life-threatening condition must be held with the upmost respect. Surgical decision making and operative technique is critical in determining the boundary of excisional debridement to perform in the absence of the ability to transfuse blood. The medical management was focused on resuscitation for sepsis, severe anemia, hyperglycemia, and wound management.

**Conclusion:**

Severely anemic patients in critical condition can survive necrotizing fasciitis with a well-planned interdisciplinary approach without compromising patient autonomy.

## Highlights

• Necrotizing fasciitis should be recognized early and transferred to an tertiary surgical center for management.
• Surgical approach should be focused on the principal of “less is more” in regards to surgical debridement technique.
• Patient values were upheld, being a Jehovah’s Witness.
• A multidisciplinary approach is critical for best prognosis.

## 1 Introduction

Necrotizing fasciitis (NF) is a soft tissue infection characterized by rapidly progressing necrosis involving the fascia and subcutaneous tissue, which can also extend into the muscle and skin [1]. Success in treatment of NF is early diagnosis, surgical intervention, and antibiotic therapy [2]. Surgical risk assessment focuses on previous undetected comorbid conditions, evaluating known comorbid conditions, and optimizing current medical conditions. Studies have demonstrated that the mortality risk increases with decreasing preoperative hemoglobin concentrations especially in patients with levels less than 6 g/dL [[3], [4], [5]]. Perioperative management strategies include establishing Jehovah’s Witness status, capacity, competence, and advanced directive [6]. We report the unique course and treatment at our academic institution of a patient who refused blood products despite having a rapidly progressing NF in the setting of sepsis, severe anemia, and having been admitted to 2 prior healthcare institutions for the same problem. This case has been reported in line with the SCARE criteria [7].

## 2 Case report

A 62-year-old man of Jehovah’s Witness faith developed a life threatening rapid progressive necrotizing fasciitis of his right lower extremity. His comorbidities consisted of type 2 diabetes mellitus, alcohol use disorder, nicotine use, and depression. He was non-compliant with his long-acting nocturnal insulin. The patient developed a non-healing right heel ulcer which began in the fall of the preceding year. He initially presented with his wound to the first health care institution 3 months later and was admitted for approximately 7 weeks. The patient subsequently underwent 4 surgical debridements and received IV antibiotics. Since that recent hospital admission, he had been following up at a local Wound Clinic.

Three weeks after discharge the patient subsequently developed increasing severe lightheadedness, generalized weakness, and dizziness which prompted patient to be evaluated in the Emergency Department (ED) of the second institution. In the ED the patient vitals were: temperature 37 °C, pulse 86 beats/min, respiratory rate 25 breaths/min, oxygen saturation of 98%, and a blood pressure of 98/59 mmHg. Physical examination was significant for right heel ulcer with purulent drainage. Initial laboratory results were significant for: WBC 32,700/mm^3^, hemoglobin 7.3 g/dL, MCV 83.4 fL, sodium 128 mmol/L, Creatinine 3.38 mg/dL, BUN 45 mg/dL, lactate 3.8 mmol/L and blood glucose level of 606 mg/dL. The patient was then admitted with septic shock, a heel ulcer with suspected osteomyelitis, and acute kidney injury. He was treated with IV antibiotics and epoetin alfa and iron sucrose parenterally. In-patient MRI of the right foot showed osteomyelitis of the calcaneus with a ruptured Achilles tendon and surrounding edema indicating infectious tenosynovitis. Subsequently during inpatient course, he developed severe right lower extremity pain and fever which prompted an emergent X-Ray of the right lower extremity. Imaging results showed prominent areas of subcutaneous emphysema throughout the soft tissue extending to the distal right femur suspicious for NF. The patient immediately underwent right-sided above the knee guillotine amputation with lateral thigh debridement. He was in critical condition post-op and a facility transfer was deemed necessary.

**Fig. 1:**
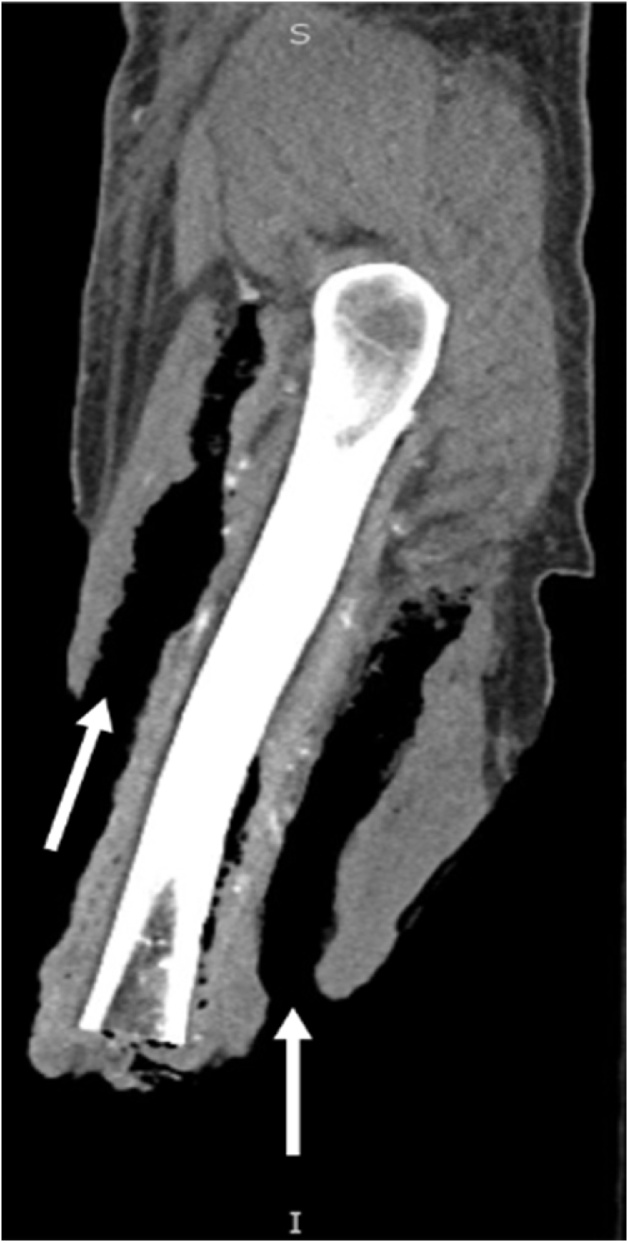
CT scan of right lower extremity. Extensive air is shown tracking superiorly within the anterior and posterior compartments of the thigh (**notated by white arrows**).

**Fig. 2:**
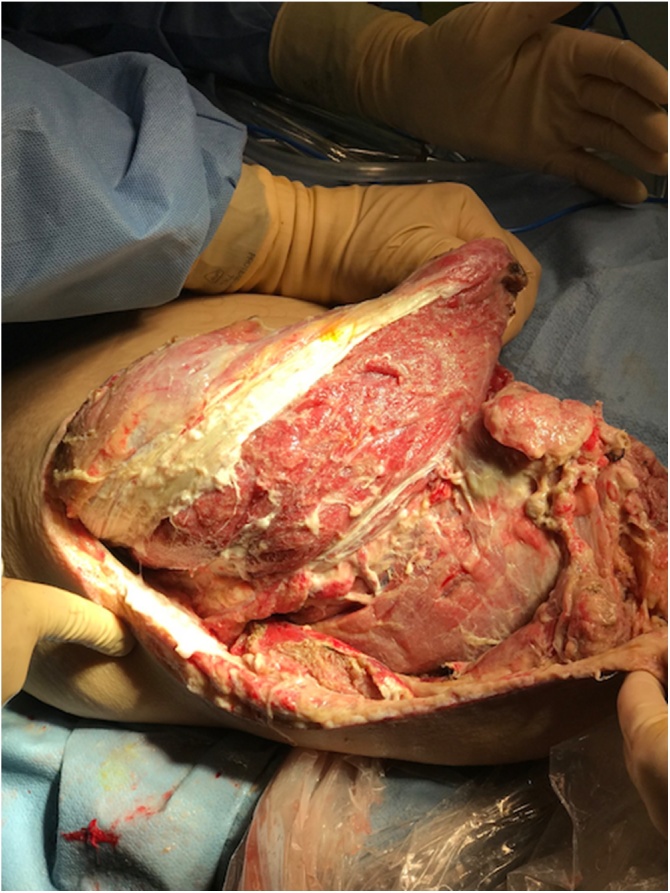
Intra-operative photo of right lower extremity. Taken during initial AKA revision and soft tissue debridement for extensive necrotizing infection.

On arrival via ambulance to the intensive care unit trauma service at the 3^rd^ institution (our hospital) the patient was in critical condition, with waxing and waning mentation, he was only oriented to self and examination revealed foul smelling AKA amputation wound which had crepitus to palpation. Significant laboratory results were: WBC of 24,800/mm^3^, hemoglobin 4.7 g/dL, MCV 75.4 fL, sodium 142 mmol/L, Creatinine 1.0, BUN 55 mg/dl, lactate 1.9 and serum glucose 300 mg/dL. The patient was resuscitated with IV fluids, IV antibiotic therapy, and pain management. He subsequently went for CT scan of the right residual limb to assess the extent of subcutaneous tissue involvement. Imaging showed subcutaneous emphysema extending to the neck of the femur (Fig. 1). Subsequently he had emergent operative wound debridement using a micro debridement tool to enhance bloodless surgery. Intraoperative discussion of a possible hip disarticulation and hemipelvectomy occurred with determination of a poor prognosis of survival. The patient therefore underwent 6 debridement surgeries over the next 14 days with the 1^st^ surgery consisting of a revision amputation (Fig. 2).

**Fig. 3:**
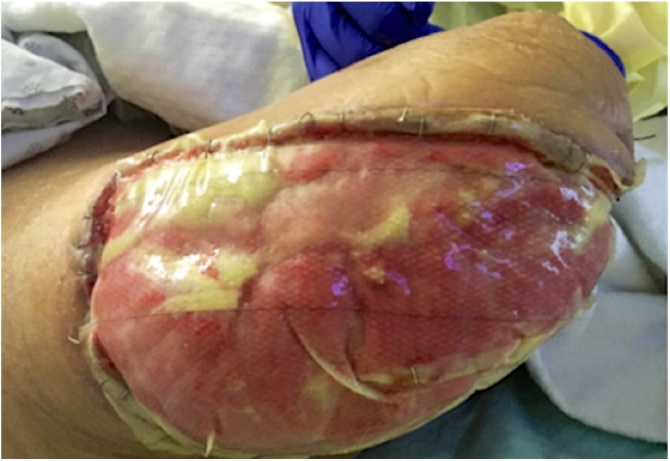
Right lower extremity wound. Integra graft over viable muscle of anterior, lateral, and posterior compartments of right thigh. Photo taken post-operative day #19 after AKA revision procedure.

Post operatively he improved significantly to a regular diet, stabilized vital signs, and normal mental status. The patient was discharged one month after initial admission to our institution to a rehabilitation facility. His wound at the time of discharge had healthy granulation tissue with a wound vac in place (Fig. 3). He was subsequently seen 2 weeks after at trauma surgeon’s office for his first outpatient follow up.

## 3 Discussion

This clinical case highlights some of the challenges involved in managing NF and the modified treatment approach used in a severely anemic patient who declines blood transfusion.

NF has a median mortality rate of 32.2%, which makes rapid diagnosis and aggressive treatment critical for the survival of the patient [8]. Early diagnoses can be challenging, and a high index of suspicion is essential. Successful treatment modalities include prompt surgical debridement and broad-spectrum antibiotics [9]. A C-reactive protein was not initially drawn and a laboratory risk indicator for necrotizing soft tissue index (LRINEC) score was not calculated however, recent systematic reviews do not recommend using LRINEC to rule out necrotizing soft tissue infection [10]. The main goals of the preoperative medical assessment were focused on sepsis and severe anemia with a hemoglobin of 4.7 g/dL on arrival to our institution. Transfusion triggers in this patient should be lower with comorbid conditions such as cardiovascular disease and sepsis. Incorporating religious beliefs into treatment plans should be discussed with the patient respectfully to make an informed choice. After refusing blood transfusion the patient agreed with utilizing aspects of bloodless medicine and was given epoetin alfa and iron sucrose perioperatively.

The patient was transferred to our facility’s trauma and acute care service for our experience in complex surgical management of soft tissue infections. The surgical procedures considered ranged from excisional debridement to hip disarticulation. These options were discussed with the patient and an informed consent was obtained. Once stabilized, the boundary on degree of surgery to perform was made perioperatively. It was felt that hip disarticulation would be non-survivable in the absence of the ability to transfuse blood. Surgical excisional debridement and revision amputation of the previous AKA was then performed. The standard surgical debridement uses scalpel/curette plus pulsed lavage, which can penetrate too deeply resulting in undesired tissue removal and/or bleeding [11,12]. Given this patient’s severe anemia and critical condition, the surgical technique involved a waterjet micro debridement instrument secondary to its precision, efficiency, and less tissue damage potential. The aim in such a case is diligent surgical technique by not over estimating the soft tissue borders with the principal of less is more. Meticulous avoidance of surgical blood loss was critical in preventing hemodynamic instability and death.

In a Jehovah’s Witness patient, the refusal of life saving therapy creates a moral dilemma for a clinician. The rights of an adult with decision making capacity in the United States to refuse medical treatment is well established [13]. The concept of autonomy honors the rights of an adult with decision making capacity thus the conversation with this patient must be held with the upmost respect. Nurses and all other members of the liaison team were also included in this conversation. The clinician must understand the trueness of the adult patient’s belief.

Timely medical decision making and management was critical in the survival of the patient. The surgical approach was designed to minimize blood loss by using a waterjet micro debridement tool. This technique avoided the pitfalls of standard excisional debridement that is too aggressive with undesirable tissue and blood loss. The medical management was focused on severe anemia, hyperglycemia and wound management. Aspects of bloodless medicine were used with the administration of erythropoietin and iron sucrose. The patient was placed on bed rest with minimal exertion and blood draws were minimized to once a week in pediatric tubes to preserve hemoglobin as frequent blood draws have been associated with hospital acquired anemia [14]. The patient’s severe hyperglycemia was controlled with an insulin drip. Post operatively vacuum-assisted closure therapy was applied to promote healing and delay subsequent skin grafting to minimize excess tissue loss [15]. The patient was very content that we upheld his beliefs and values; this was crucial to ensure patient cooperation and shared decision-making.

## 4 Conclusion

This case demonstrates the complexity involved in managing a severely anemic and septic Jehovah’s Witness patient with NF. Such a patient should be recognized and transferred to a trauma and acute care center, well known and reputable for the institutions experience and exposure to complex cases. A multidisciplinary approach, discussion, and plan of care should be held with all stakeholders. The surgical technique with the principal that less is more is particularly valuable while managing a case like this. The aim is to be more precise in the margin of error regarding surgical excision. Necrotizing fasciitis, sepsis, and severe anemia in a Jehovah’s Witness patient with hemoglobin of 4.7 g/dL is not necessary a lethal condition.

## Funding

No sources of funding.

## Ethical approval

Ethical review/ approval by Medical Institution is not required, as long as there is a documented and signed consent by the patient.

## Consent

Written informed consent was obtained from the patient for publication of this case report and accompanying images. A copy of the written consent is available for review by the Editor-in-Chief of this journal on request.

## Author contribution

Sanjeev Kaul MD: Study conception, Design, Acquisition of data, Analysis and interpretation of data, Drafting of manuscript, and Critical Revision.

Chinwe Ogedgbe MD MPH: Acquisition of data, Analysis and interpretation of data, drafting of manuscript, and critical revision.

Jayson Fernando MSc: Acquisition of data, analysis and interpretation of data, and drafting of manuscript.

## Registration of research studies

N/A Case Report.

## Guarantor

Sanjeev Kaul, MD.

## Provenance and peer review

Not commissioned, externally peer-reviewed.

## Declaration of Competing Interest

No conflicts of interest, by all 3 authors.
